# Comparison of biological effects of modulated electro-hyperthermia and conventional heat treatment in human lymphoma U937 cells

**DOI:** 10.1038/cddiscovery.2016.39

**Published:** 2016-06-13

**Authors:** G Andocs, M U Rehman, Q-L Zhao, Y Tabuchi, M Kanamori, T Kondo

**Affiliations:** 1 Department of Radiological Sciences, Graduate School of Medicine and Pharmaceutical Sciences, University of Toyama, Toyama, Japan; 2 Division of Molecular Genetics, Life Science Research Center, University of Toyama, Toyama, Japan; 3 Department of Human Science, Graduate School of Medicine and Pharmaceutical Sciences, University of Toyama, Toyama, Japan

## Abstract

Loco-regional hyperthermia treatment has long history in oncology. Modulated electro-hyperthermia (mEHT, trade name: oncothermia) is an emerging curative treatment method in this field due to its highly selective actions. The impedance-matched, capacitive-coupled modulated radiofrequency (RF) current is selectively focused in the malignant cell membrane of the cancer cells. Our objective is studying the cell-death process and comparing the cellular effects of conventional water-bath hyperthermia treatment to mEHT. The U937 human histiocytic lymphoma cell line was used for the experiments. In the case of conventional hyperthermia treatment, cells were immersed in a thermoregulated water bath, whereas in the case of mEHT, the cells were treated using a special RF generator (LabEHY, Oncotherm) and an applicator. The heating dynamics, the maximum temperature reached (42 °C) and the treatment duration (30 min) were exactly the same in both cases. Cell samples were analysed using different flow cytometric methods as well as microarray gene expression assay and western blot analysis was also used to reveal the molecular basis of the induced effects. Definite difference was observed in the biological response to different heat treatments. At 42 °C, only mEHT induced significant apoptotic cell death. The GeneChip analysis revealed a whole cluster of genes, which are highly up-regulated in case of only RF heating, but not in conventional heating. The Fas, c-Jun N-terminal kinases (JNK) and ERK signalling pathway was the dominant factor to induce apoptotic cell death in mEHT, whereas the cell-protective mechanisms dominated in case of conventional heating. This study has clearly shown that conventional hyperthermia and RF mEHT can result in different biological responses at the same temperature. The reason for the difference is the distinct, non-homogenous energy distribution on the cell membrane, which activates cell death-related signalling pathways in mEHT treatment but not in conventional heat treatment.

## Introduction

### What is modulated electro-hyperthermia (mEHT)?

mEHT (trade name: oncothermia) is an electromagnetic heat treatment method, a non-invasive cellular selective oncotherapy, using the capacitive-coupled energy of 13.56 MHz radiofrequency (RF) to destroy the malignant cells. It was introduced into the human oncological treatment practice more than 20 years ago, and since then its therapeutic benefits have been proven in many different areas of clinical oncology.^1–5^ In parallel with the clinical application, intensive basic research has been performed to get a better understanding of the underlying cellular and molecular biology effects of the RF-field interaction with living tissue.^6,7^ In previous *in vivo* studies, it was observed that mEHT treatment can induce massive programmed cell death in the treated tumour,^8^ and this apoptotic cell death process has some unique immunological aspects,^9^ which can open up possible new immunotherapeutic combination modalities.^10,11^

### Theoretical background

In one of our previous investigations, a comparative *in vivo* study was performed to reveal the difference in the biological response between conventional heat treatment and mEHT.^12^ In this experiment, the RF heating induced much more significant tumour tissue distraction, even in a physiological temperature range, than conventional heat treatment. This unique characteristic of the RF heating was realised many decades ago by different workgroups; however, the exact explanation of this effect is still missing. There are several theoretical considerations about the existence of a special non-thermal effect of the RF field,^13–15^ but these remain controversial,^16^ and still lack unequivocal experimental evidence and a widely accepted explanation of its mechanism of action.^17^

Another interesting approach to explain the special nature of the biological effect of the RF field is the so-called microthermal concept. This hypothesis was first introduced by Lebovitz,^18^ and since then many research groups have proved that RF exposure of biological material (cells) would induce a highly non-homogenous energy distribution on the cell membrane.^19–21^ Unfortunately, these models and studies did not take into considerations, which was achieved in the past few years in connection with the fine microstructure of the cell membrane.

The classic fluid mosaic membrane model^22^ became outdated after recent research results had been revealed that the cell membrane has a highly organised microstructure, comprising special microdomains of the membrane, called membrane rafts.^23,24^ According to Pike: ‘Lipid rafts are small (10–200 nm), heterogeneous, highly dynamic, sterol- and sphingolipid-enriched domains that compartmentalise cellular processes. Small rafts can sometimes be stabilised to form larger platforms through protein–protein and protein–lipid interactions’.^25^ A variety of proteins, especially those involved in cell signalling, have been shown to partition into lipid rafts. As a result, lipid rafts are thought to be involved in the regulation of signal transduction.^26^ Although rafts have a distinctive protein and lipid composition, it is obvious that its electromagnetic parameters (dielectric constant, permittivity, conductivity, etc.) differ from the other part of the cell membrane.

**Figure 1:**
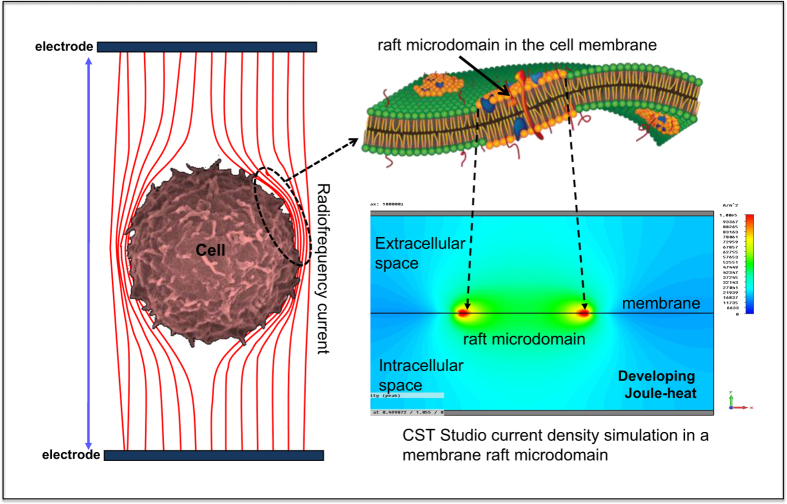
Schematic representation of the membrane raft-specific, localised heat stress.

According to our knowledge, our workgroup studied the biological effect of the RF field for the first time, taking into consideration the lipid raft membrane model,^27^ developed a well-elaborated theoretical framework of capacitive-coupled RF field interaction with cell membrane microdomains,^28^ and performed a computer simulation and *in vitro* experiments to prove the hypothesis.^29^ The theory is described in detail elsewhere,^30^ but very briefly below see Figure 1.

The capacitive-coupled RF current at 13.56 MHz frequency and in the applied field strength (<500 V/m) cannot penetrate into the cell interior, because the cell membrane is an excellent electric insulator.^31^ The energy of the amplitude-modulated RF current is absorbed by the extracellular matrix,^32^ and mostly the cell membrane by beta-dispersion of the plasma membrane.^33^ The membrane rafts, according to their different electromagnetic properties, absorb more RF power than the other lipid bilayer part of the cell membrane. The higher current density and dielectric loss of the membrane rafts was proven by special software simulation.^34^ The higher energy absorption means higher local temperature of the raft microdomain during continuous RF current delivery to the cell.

### The purpose of the study

In our previous investigations, we found that mEHT treatment can induce the same biological response (apoptotic cell death) in U937 cell line at 3 °C lower than conventional water bath hyperthermia (WHT) treatment.^29^ According to previous reports from different workgroups, 42 °C heat treatment for 30 min induced no cell damage in this cell line.^35–38^ In the present study, we compared the biological effect of mEHT treatment and conventional WHT treatment in the U937 cell line at 42 °C, and investigated the underlying molecular mechanism of the induced-biological response.

## Results

### mEHT-induced apoptosis

**Figure 2:**
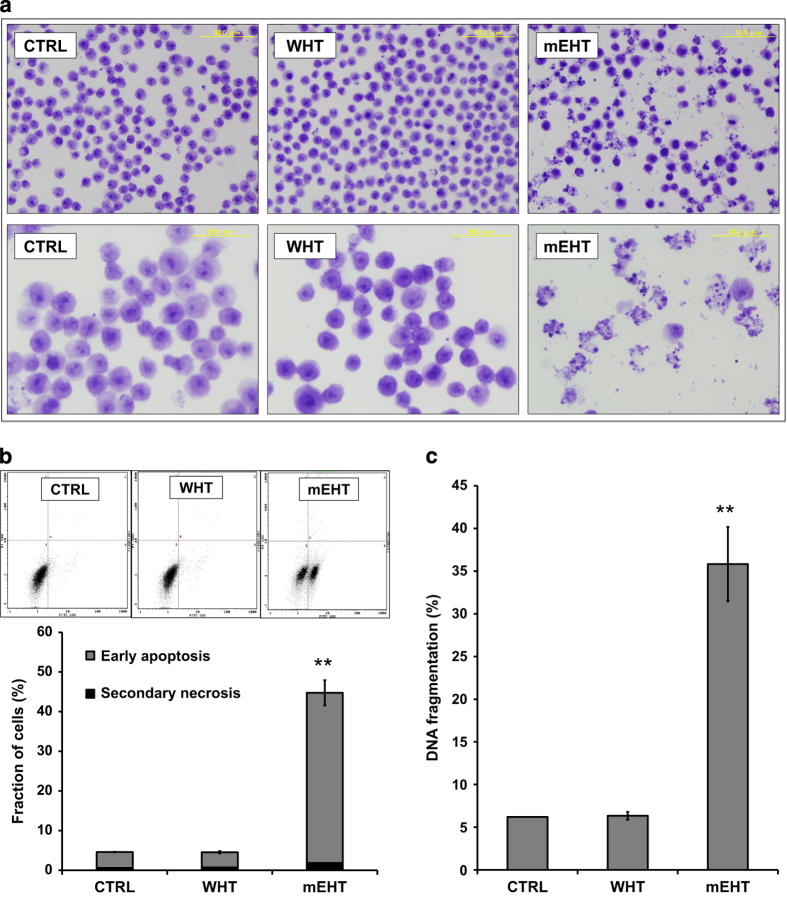
Representative microscopy images of untreated control, WHT-treated and mEHT-treated cells, 3 h post-treatment in Giemsa stained cell samples (**a**). Representative flow cytometric histogram of the Annexin V-FITC and PI staining 3 h after treatment. The quantitative analysis shows a significant increase of Annexin V positive-cell fraction only in mEHT samples (**b**). Quantitative results from DNA fragmentation assay. The mEHT-treated cells showed a significant increase in DNA fragmentation percentage, which is one of the hallmarks of apoptotic cell death (**c**). The results are presented as the mean±S.D. (*n*=3). **P<0.01 as compared to WHT treatment.

The morphological changes evident of apoptosis were detected using bright field microscopy imaging in fixed and Giemsa stained cell samples (Figure 2a) showed that the typical morphological changes associated with apoptosis were more prominent in the mEHT-treated cells than in WHT-treated cell samples. To further confirm the apoptosis induction after 3 h of treatment, cells were subjected to the DNA fragmentation assay. It was found that the mEHT treatment showed significant enhancement of DNA fragmentation percentage compared to WHT treatment (Figure 2c), whereas WHT did not induce an increase in DNA fragmentation percentage.

Further, to distinguish between early and late phase apoptosis (secondary necrosis), cells were subjected to Annexin V-FITC/PI double staining. Flow cytometry analysis revealed that mEHT treatment significantly enhanced the percentage of early apoptotic (Annexin V-FITC) cells (apoptotic percentage), compared with treatment WHT alone (Figure 2b).

### mEHT-induced loss of mitochondrial membrane potential (MMP)

**Figure 3:**
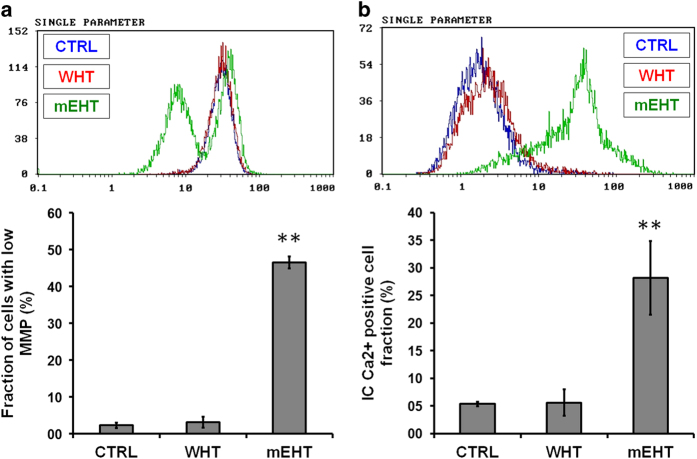
Representative flow cytometric histogram of the MMP. The quantitative analysis revealed a significantly higher number of cells with low MMP in mEHT-treated cells (**a**). Representative flow cytometric histogram of the intracellular Ca^2+^ measurement. The quantitative analysis revealed significantly higher intracellular Ca^2+^ level in mEHT-treated cells (**b**). The results are presented as the mean±S.D. (*n*=3). **P<0.01 as compared to WHT treatment.

To assess the role of mitochondrial function in the mEHT-induced apoptosis, its effect on MMP was evaluated. The fraction of cells with low MMP significantly increased in the mEHT treatment compared to either control or WHT alone (Figure 3a).

### Effects of mEHT on intracellular [Ca^2+^]_i_

As U937 cells have been known to possess a [Ca^2+^]_i_ dependent apoptosis pathway, their role in mEHT-induced apoptosis was investigated. Cells were treated with mEHT and WHT; after 3 h of incubation, changes in the [Ca^2+^]_i_ were detected by flow cytometry using Fluo-3-AM dye. The mEHT treatment significantly increased the [Ca^2+^]_i_ concentration, whereas no such change was observed in WHT treatment alone (Figure 3b).

### Effects of mEHT on the MAPK pathway

**Figure 4:**
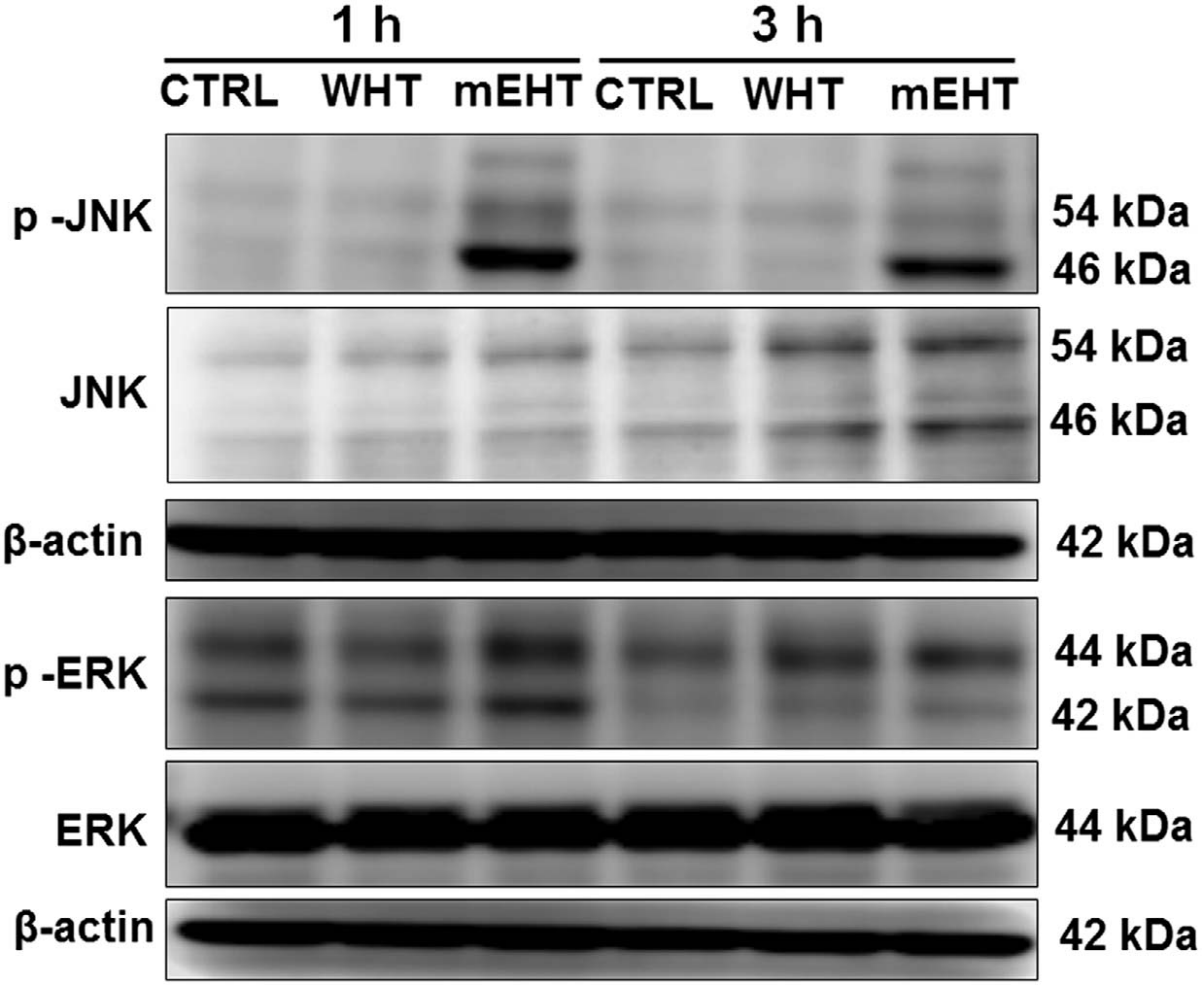
Expression of p-JNK was increased at 1 and 3 h, similarly p-ERK was also increased at 1 h following mEHT treatment as evidenced by the WB analysis.

In order to investigate the effects of mEHT treatment on the MAPK pathway, western blot (WB) analysis was performed at 1 and 3 h after mEHT and WHT treatment. The results showed that the phosphorylation of JNK was markedly increased following mEHT treatment at 1 and 3 h. Similarly, at 1 h phosphorylation of ERK was also more increased with mEHT treatment than the WHT treatment. However, no significant change was observed in the full form of JNK and ERK with both treatments (Figure 4).

### FAS externalisation and caspase-8 activation induced by mEHT treatment

**Figure 5:**
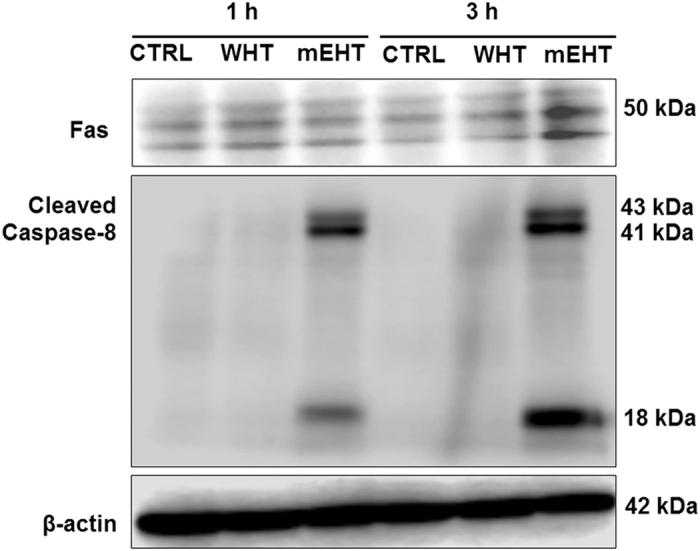
Western blot analysis revealed increased FAS expression at 3 h; subsequently the expression of cleaved caspase-8 was also markedly increased at 3 h following mEHT treatment.

The Fas receptor is a death receptor on the surface of cells that leads to one of the apoptotic pathways, the extrinsic pathway, through death-inducing signalling complex (DISC) assembly and subsequent caspase-8 activation. To determine the involvement of the extrinsic apoptotic pathway, the effects of mEHT treatment on Fas externalisation and caspase-8 activation were studied. The protein expression of Fas was markedly increased 3 h after mEHT treatment compared to treatment with WHT alone. Simultaneously, increased cleaved caspase-8 was observed after 1 and 3 h following mEHT treatment than the WHT treatment (Figure 5).

### Expression of apoptosis-related proteins

**Figure 6:**
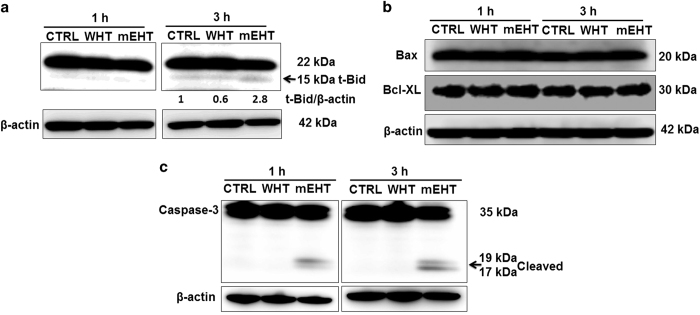
The expression of pro-apoptotic Bcl-2 family protein, truncated Bid (t-Bid, an active form of Bid, indicated by the arrows) was increased following mEHT treatment at 3 h. The t-Bid signals were normalised to the *β*-actin signals, and the relative ratios are shown below each band (**a**). However, expression of Bax and Bcl-XL remained unchanged (**b**). The WB analysis clearly showed the increased expression of cleaved caspase-3 in mEHT-treated samples at 1 and 3 h (**c**).

Bcl-2 family proteins with anti- or pro-apoptotic functions have a pivotal role in apoptosis by releasing cytochrome-*c* from the mitochondria, to activate the caspase cascade. To investigate the involvement of Bcl-2 family proteins in the mEHT treatment-induced apoptosis, WB analysis was undertaken. Increased expression of pro-apoptotic Bcl-2 family protein, truncated Bid (t-Bid, an active form of Bid) was observed following mEHT treatment. However, activation of Bid with WHT was not observed (Figure 6a), and the expression of pro-apoptotic Bax and anti-apoptotic Bcl-XL remain unchanged (Figure 6b).

As caspases are considered the main executioner of apoptosis, the effect of mEHT treatment on caspase-3 was evaluated. The active form of caspase-3 (cleaved capase-3) was markedly increased with mEHT, whereas no increase was observed with WHT (Figure 6c).

### Gene chip analysis

**Figure 7:**
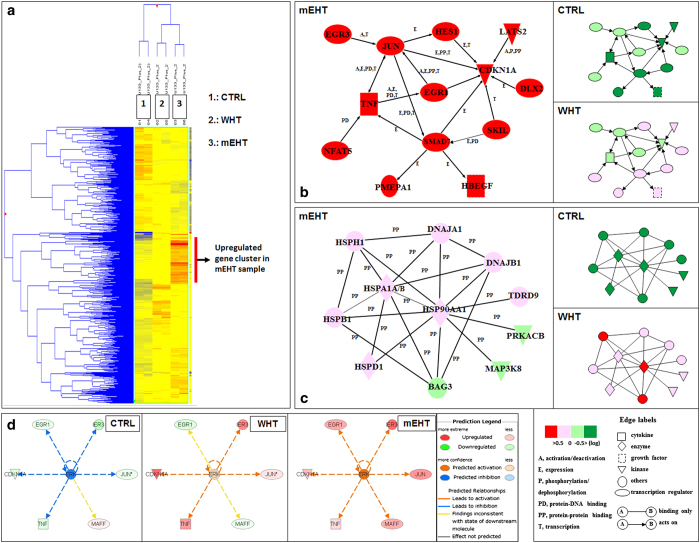
The GeneChip heat map. The analysis revealed a whole cluster of genes, which are highly up-regulated in the case of mEHT-treated samples, but not in WHT (**a**). The ingenuity pathway analysis clearly demonstrated a specific gene network containing cell death-related genes, such as EGR1, JUN, and CDKN1A. The expression levels of these genes were elevated only in the cells treated with mEHT (**b**). A specific cytoprotective gene network (heat shock proteins, HSPs) was activated in the case of WHT treatment, but not in mEHT-treated samples (**c**). The ingenuity pathway analysis predicted ERK activation and the relationship between JUN and ERK in OTM-treated cells. This activation mechanism is missing in the case of WHT-treated cells (**d**).

To identify the biological functions and gene networks regulated by mEHT treatment, we carried out gene-expression profiling coupled to functional and signalling characterisation by the Ingenuity software Inc. (Mountain View, CA, USA). Global-scale gene expression analysis of cells treated with mEHT identified a whole cluster of genes that were up-regulated by a factor of 2 or greater (Figure 7a). In addition, functional category and gene network analyses were conducted by use of the Ingenuity Pathways Knowledge Base (Ingenuity Systems Inc., Mountain View, CA, USA). According to the careful analysis, two markedly different activated gene networks were identified (Figures 7a and b) in mEHT and WHT samples, respectively. In gene network A, containing the up-regulated genes in mEHT-treated cells, this was mainly associated with the molecular function of cell death (EGR1, JUN, and CDKN1A). In WHT-treated cells, however, this cell-death related gene network remained silent. In gene network B containing the up-regulated genes in WHT-treated cells, several HSPs, such as Hsp105 (HSPH1), Hsp90A (HSP90AA1) were observed. This cytoprotective gene network B remained silent in mEHT-treated cells. The ingenuity pathway analysis predicted ERK activation and the relationship between JUN and ERK in OTM-treated cells. This activation mechanism is missing in the case of WHT-treated cells (Figure 7d).

## Discussion

Our results have clearly demonstrated the significant difference in the biological response of different forms of heat treatments, but at the same temperature in U937 hystiocytic lymphoma cell line. mEHT treatment at 42 °C temperature level induced massive apoptotic cell death, showing the characteristic morphological signs of apoptosis 3 h post-treatment in Giemsa stained samples. The quantitative analysis also showed a pronounced increase of apoptotic cell fraction in Annexin V-FITC/PI assay. Massive DNA fragmentation was also measured in the mEHT-treated cell samples. None of these signs of cell death were observed in WHT-treated cell samples at 42 °C. This result has a good correspondence with the findings reported in previous papers.^35,39^

But what can be the reason of this huge difference between the two treatments when the temperature level of both treatment methods was the same?

### Activation of JNK pathway

The findings of the WB analysis are in line with the gene chip analysis results. The gene chip analysis predicted the ERK and JNK pro-apoptotic pathway activation in mEHT treatment (Figure 7d), and we have also found a significant p-ERK and p-JNK level elevation in the mEHT-treated samples by the WB analysis. (Figure 4). This pathway was not activated in WHT-treated samples. Previous studies reported that hyperthermia treatment is a strong activator of JNKs^35,39^ but its activation by heat stress requires a temperature of at least 44 °C. JUN activation was also observed by Furusawa *et al.*^39^ at 44 °C. It is very interesting that mEHT treatment-induced the same JNK pathway activation at 42 °C where conventional heat treatment cannot induce similar changes. This observation supports our theory of the selective membrane raft heating by the applied RF current, because the heat-stressed cell membrane might have been a good activator of the JNK pathway; however, the upstream signalling mechanism is not yet completely understood.^40^ There are some interesting observations, which suppose strong connections between membrane raft-mediated signalling and JNK activation.^41,42^

### Activation of cytoprotective gene network in WHT samples

The gene chip analysis revealed a highly cytoprotective gene network activation in the WHT samples. This network contains up-regulated HSP genes, such as Hsp105 or Hsp90A. HSPs (molecular chaperons) have been shown to block apoptosis by interfering with caspase activation and to inhibit apoptosis in a direct or indirect manner.^43,44^ HSPs under the present experimental conditions appeared to behave as anti-apoptosis molecules and prevent any apoptotic cell death in WHT-treated cells. This cytoprotective gene network remained silent in mEHT-treated cells, hence could not block the activated caspase cascade.

### Activation of death receptor signalling pathways

We found massive Fas receptor up-regulation in mEHT-treated samples (Figure 5). Fas (CD95 or APO-1) is considered to be the prototypic and major member of the death-receptor family, a subgroup of the TNF receptor superfamily, which also includes the TRAIL receptors TRAIL-R1 (DR4) and TRAIL-R2 (DR5) and can transmit apoptotic signals through the presence of a cytoplasmic death domain.^45,46^ The Fas receptor can be activated by its ligand (FasL). The U937 cells can express FasL, so autocrine Fas-mediated apoptotic cell death mechanism is possible in our case.

However, there is a more interesting possibility for the activation of Fas and other death receptors, which does not require the death receptor ligand.

There are several reports from the past years that death receptors, and especially Fas, are the most abundant transmembrane receptors in the membrane raft domains.^47–49^ A growing amount of evidence points to the notion that membrane rafts can serve, in addition to generate a high local concentration of Fas, as platforms for coupling adaptor and effector proteins required for Fas signalling.^47,50^ This is of particular significance in Fas-mediated signal transduction, as the initial signalling events depend on protein–protein interactions. Furthermore, this could facilitate and amplify signalling processes by local assembly of various cross-interacting signalling molecules.^51^

Gajate and Mollinedo and their workgroup made a pioneering work to elucidate the molecular mechanism of membrane raft remodelling in Fas-mediated apoptotic signalling. They stated: ‘Modulation of survival and apoptotic signalling routes via rafts could be a promising approach in cancer therapy, and growing evidence shows the potential of rafts as therapeutic targets in cancer therapy.^52,53^ In this regard, the formation of the apoptotic signalling-rich raft clusters named CASMERs^50,54^ favours the onset of apoptosis by bringing together death-promoting receptors and downstream signalling in a rather limited space, thus facilitating protein interactions and the triggering and launching of a potent apoptotic response'.^55^

Alterations in the plasma membrane fluidity and lipid rafts have often been found to be linked during the course of apoptosis. Specific modification of the membrane raft microstructure, especially by increasing fluidity of the membrane and the rafts using different chemical agents (e.g. edelfosine,^56^ cisplatin,^57^ ethanol,^58^ resveratrol^59^) result in apoptotic cell death in various experimental conditions in various model systems.^60^

There is also a growing number of evidence that heat shock and certain hyperthermic conditions (i.e. fever) can alter the membrane raft microdomains leading to the activation of death receptors and apoptotic cell death.^61–64^ Heat treatment can also increase the fluidity of the membrane and the raft, initialising different signal transduction pathways including caspase cascade signalling.^65^

According to our theory, alterations of the membrane raft can be done without any specific chemical agent or drug; this can be achieved by biophysical manipulation (specific RF energy deposition and consequent heat stressing) of the membrane rafts of the cancer cells.

**Figure 8:**
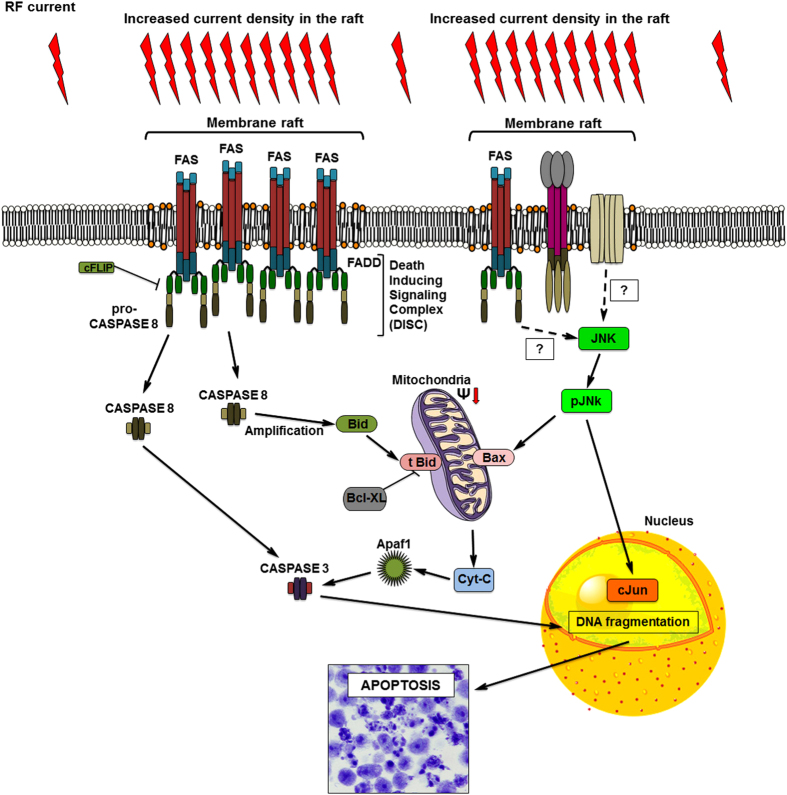
Schematic summary of the proposed molecular mechanism.

On the basis of our initial hypothesis, the membrane raft can absorb more RF energy than the other part of the membrane. The reason of this increased RF absorption rate is the different electromagnetic properties (electrical conductivity and permittivity) of the membrane raft, due to its different chemical composition, comparing to the simple membrane bilayer. This difference was proven by precise computer simulation.^29,34^ If we accept this theory, our experimental findings can be explained in a simple way (Figure 8).

The increased RF energy absorption of the membrane raft heated it up, resulting in localised heat stress. The heat on the membrane raft increased its fluidity and similarly to heat treatments modified and remodelled the membrane raft microstructure, activating the death receptor Fas signalling pathway. Activation of Fas leads to the recruitment of the adaptor molecule Fas-associated death domain protein and procaspase-8, forming the so-called DISC,^66^ containing Fas, Fas-associated death domain and procaspase-8 molecules. Fas aggregation and DISC formation result procaspase-8 transactivation that releases the active caspase-8, thus initiating apoptosis through a subsequent caspase cascade, activating the effector caspases (caspase-3).^67,68^ Furthermore, Fas can also trigger the intrinsic (mitochondrial) apoptotic cell death pathway through cleavage of BH3-interacting domain death agonist (Bid) into truncated-Bid (t-Bid).^69–72^ Consistent with this, we found that the mEHT-treated samples showed caspase activation and significant loss of MMP, with increased [Ca^2+^]_i_, release. The activation of the extrinsic and intrinsic apoptotic signalling pathway resulted massive apoptotic cell death in mEHT-treated cells.

In this study, we proposed a novel mechanism for the biophysical manipulation of the membrane raft microdomain of the cancer cell using properly applied mEHT treatment. The specific RF energy absorption of the membrane raft results in distinct, inhomogenous energy distribution on the cell membrane causing localised heat stress, which activates cell death-related signalling pathways in mEHT treatment but not in conventional WHT treatment.

## Materials and methods

### Cell culture

U937, a human myelomonocytic lymphoma cell line from Human Sciences Research Resource Bank (Japan Human Sciences Foundation, Tokyo, Japan) was used for the experiments. The cells were grown in RPMI 1640 culture medium supplemented with 10% heat-inactivated foetal bovine serum at 37 °C in humidified air with 5% CO_2_. Cells were sub-cultured every second day, and were used for the experiments in their log phase. Cells were treated in 10^6^ cells/ml density at a total volume of 8 ml.

### Treatment processes

Here, 8 ml U937 cell suspension was used in both the treatment objects. In the mEHT treatment process, the suspension was pipetted into a coverslip-bottomed slide-flask (Nunc Lab-Tek II Chambered Coverglass, Thermo Fisher Scientific, Inc., Waltham, MA, USA) and placed into a special custom-designed platinum electrode applicator. (See the Supplementary material for details.) The active RF-electrode pure platinum (99.9%) was used to minimise electrode by-products. The active electrode was immersed in the cell suspension. The effective surface of this platinum electrode was 10mm×45mm. The AM modulated 13.56 MHz RF source (LabEhy100, Oncotherm Ltd., Troisdorf, Germany) was connected to the applicator via a precise impedance matching unit. The complete heating-time was 30 min.

In case of conventional WHT, 8 ml cell suspension was filled in a 15 ml centrifuge tube and then placed into a thermoregulated water bath (Thermo Minder SD Mini, Taitek Corp., Tokyo, Japan) continuously measuring the temperature profile during the treatment. The complete heating-time was well fit to mEHT, 30 min each. See Supplementary Figure 1 for the detailed experimental setup.

The heating dynamics and the treatment time at maximum temperature were the same in all treatments. After the treatments, cell suspensions were placed back into 10 cm plastic Petri dishes (BioBik, Ina-Optika Co. Ltd., Osaka, Japan) and incubated for 24 h. All experiments were performed in triplicate.

### Temperature measurement during the heat treatments

The temperature change of the cell suspension during the treatments was carried out by a four channel fluoroptic temperature measurement system (Luxtron m3300 Biomedical Lab Kit, Lumasense Technologies, Santa Clara, CA, USA). The temperature measurement probe is 0.5 mm in diameter and is an optical fibre, which is totally insensitive for the electromagnetic field. Probes 1 and 2 were used to monitor the temperature changes in the case of conventional heating, and probes 3 and 4 were used to measure the temperature profile of the mEHT treatment. The probes were precisely positioned to the inner surface of the slide-flask as well as to the lowest point in the centrifuge tube. The measured temperature parameters were recorded real-time (1 sampling per second) in a PC. A representative temperature measurement graph can be seen in the Supplementary Figure 2.

### Morphological detection of apoptosis

To identify the morphological changes of the apoptotic cells after mEHT and conventional heat treatments, the cells were examined by Giemsa staining.

Cells were collected after 3 h of incubation at 37 ºC, washed with PBS and collected by centrifugation. Then, the cells were fixed with methanol and acetic acid (3:1) for 24 h and spread onto glass slides. After drying, staining was performed with 5% Giemsa solution (pH 6.8) for 20 min, then washed with tap water. The cell samples on the slides were covered with a coverslip using Eukitt (O. Kindler GmbH & Co., Freiburg, Germany). Cells were imaged using a conventional bright field microscope (Olympus BX61 Olympus Corp., Tokyo, Japan) equipped with a standard microscope camera (Olympus DP70, Olympus Corp., Tokyo, Japan).

### Detection of apoptosis using Annexin V-FITC/PI staining

To quantitatively investigate the different heat treatment-induced early apoptosis and secondary necrosis, phosphatidylserine externalisation of apoptosis was determined by analysis of PI and fluorescein isothiocyanate-labelled Annexin V (Immunotech, Marseille, France) using Flow cytometry (Epics XL, Beckman-Coulter, Miami, Florida, USA)^73^ according to the instructions of the manufacturer. Briefly, followed by RF and conventional heat treatment, cells were collected after 3 h of 37 °C incubation, washed with cold PBS at 4 °C and centrifuged at 1200 r.p.m. for 3 min. The resulting pellet was mixed with binding buffer of the Annexin V-FITC kit. FITC-labelled Annexin V (5 *μ*l) and PI (5 *μ*l) were added to the 490 *μ*l suspension and mixed gently. After incubation at 4 °C for 20 min in the dark, the cells were analysed by flow cytometry.

### DNA fragmentation assay

For the detection of apoptosis, the percentage of DNA fragmentation was assessed until 3 h post-treatment using the method of Sellins and Cohen,^74^ with minor modifications. Briefly, ~3×10^6^ cells were lysed using 200 *μ*l of lysis buffer (10 mM Tris, 1 mM EDTA and 0.2% Triton X-100, pH 7.5) and centrifuged at 13 000×*g* for 10 min. Subsequently, each DNA sample in the supernatant and the resulting pellet was precipitated in the 25% trichloroacetic acid at 4 °C overnight and quantified using a diphenylamine reagent after hydrolysis in 5% trichloroacetic acid at 90 °C for 20 min. The percentage of fragmented DNA in each sample was calculated as the amount of DNA in the supernatant divided by total DNA for that sample (supernatant plus pellet).

### Measurement of MMP

After 3 h of incubation post-treatment at 37 °C, cells were harvested and stained with 10 nM tetramethylrhodamine methyl ester (Molecular Probes, Eugene, OR, USA) for 15 min at 37 °C in 1 ml of PBS, followed by the immediate flow cytometry of red TMRM fluorescence (excitation at 488 nm; emission at 575 nm).

### Measurement of [Ca^2+^]_i_

To assess the effects of combined treatment on intracellular calcium homeostasis, intracellular free [Ca^2+^]_i_ was measured using calcium probe Fluo-3/AM (Dojindo Laboratories Co., Ltd., Kumamoto, Japan). After 3 h of post-treatment incubation at 37 °C, the cells were collected and then loaded with 5 *μ*M Fluo-3/AM for 30 min at 37 °C. Excess Fluo-3/AM was removed by washing three times with PBS. The fluorescence intensity of free [Ca^2+^]i levels was measured by flow cytometry.

### Microarray and computational gene expression analyses

Gene expression was analysed using a GeneChip system with a Human Genome U133-plus 2.0 array, which was spotted with ~54 000 probe sets (Affymetrix Inc., Santa Clara, CA, USA). Samples for array hybridisation were prepared as described in the Affymetrix GeneChip Expression Technical Manual (Affymetrix Inc.). The scanned arrays were analysed using the GeneChip Analysis Suite Software (Affymetrix Inc.). The obtained hybridisation intensity data were analysed using the GeneSpring analysis software (Silicon Genetics, Redwood City, CA, USA) to extract the significant genes. To examine gene ontology, including the biological processes, cellular components, molecular functions, and gene networks, the obtained data were analysed using the Ingenuity Pathways Analysis tools (Ingenuity Systems Inc.), a web-delivered application that enables the identification, visualisation, and exploration of molecular interaction networks in gene-expression data.

### Western blot analysis

The cells were collected and washed with cold PBS. Cells were lysed at a density of 2.0x10^6^ cells/100 *μ*l of RIPA buffer (50 mM Tris–HCl, 150 mM NaCl, 1% Nonidet P-40 (v/v), 1% sodium deoxycholate, 0.05% SDS, 1 *μ*g of each aprotinin, pepstatin and leupeptin and 1 mm phenylmethyl sulphonyl fluoride) for 20 min. Following brief sonification, the lysates were centrifuged at 12 000×*g* for 10 min at 4 °C, and the protein content in the supernatant was measured using the Bio-Rad protein assay kit (Bio-Rad, Hercules, CA, USA). Protein lysates were denatured at 96 °C for 5 min, after mixing with SDS-loading buffers, applied on an SDS-PAGE gel (Daiichi Pure Chemicals Co., Ltd, Tokyo, Japan) for electrophoresis, and transferred to a nitrocellulose membrane (Amersham Biosciences, Buchinghamshire, UK). Western blot analysis was performed to detect Caspase-3, cleaved caspase-8, Bid, Bax, Bcl-2, Fas, JNK, P-JNK, ERK, P-ERK, and *β*-actin expression using specific polyclonal antibodies. Blots were then probed with either secondary horseradish peroxide-conjugated anti-rabbit or anti-mouse IgG antibodies obtained from Cell Signalling. Band signals were visualised on a luminescent image analyser (LAS 4000, Fujifilm Co., Tokyo, Japan) using chemiluminescence ECL detection reagents (Amersham Biosciences). Because of the weak visibility of the t-Bid signal, band density was quantified in this case by Image Studio software version 4 (LI-COR Biosciences, Lincoln, NE, USA), and the relative amounts of proteins associated with specific antibodies were normalised according to the intensities of *β*-actin.

### Statistical analysis

The values are expressed as the means±SD. Statistical significance differences were evaluated using the Student’s *t*-test. Values of *P*<0.05 were considered to be significant. All experiments were performed at least in triplicate.
