# Supplementary material for: Comparison of biological effects of modulated electro-hyperthermia and conventional heat treatment in human lymphoma U937 cells
Source: Cell Death Discov. 2016 Jun 13;2:16039–. doi: 10.1038/cddiscovery.2016.39 (PMC4979466; doi:10.1038/cddiscovery.2016.39)
Supplement: Supplementary Information [file cddiscovery201639-s1.doc]

Supplementary information


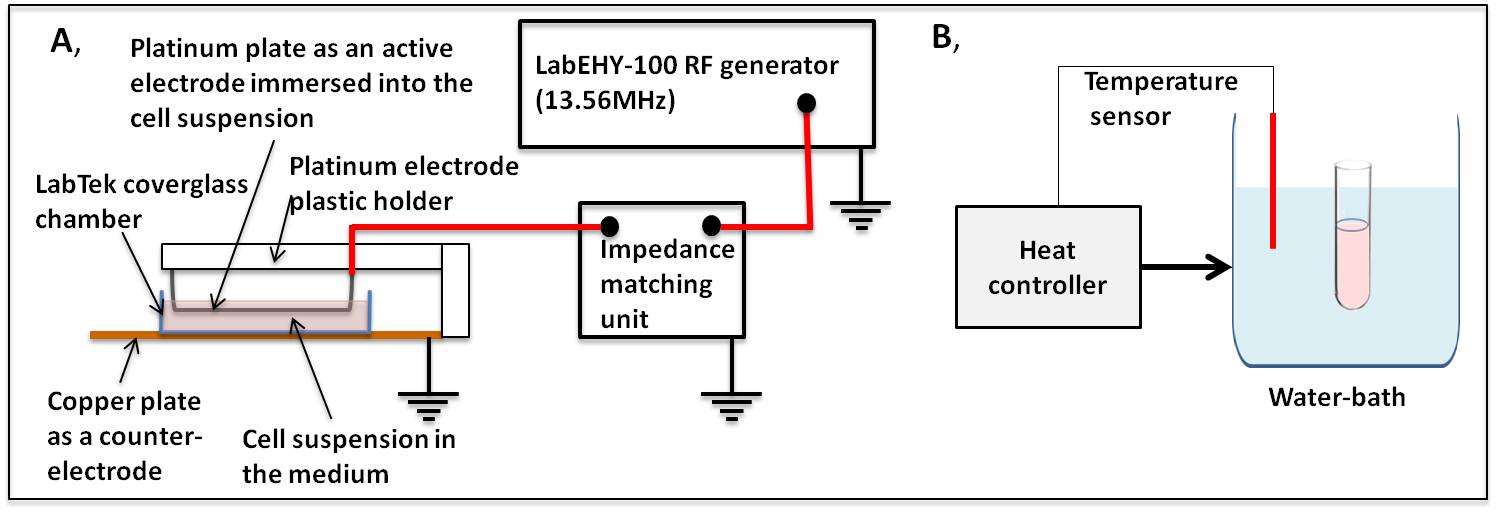


Figure 1. The experimental setups of the hyperthermia treatments. mEHT (A) and WHT (B)


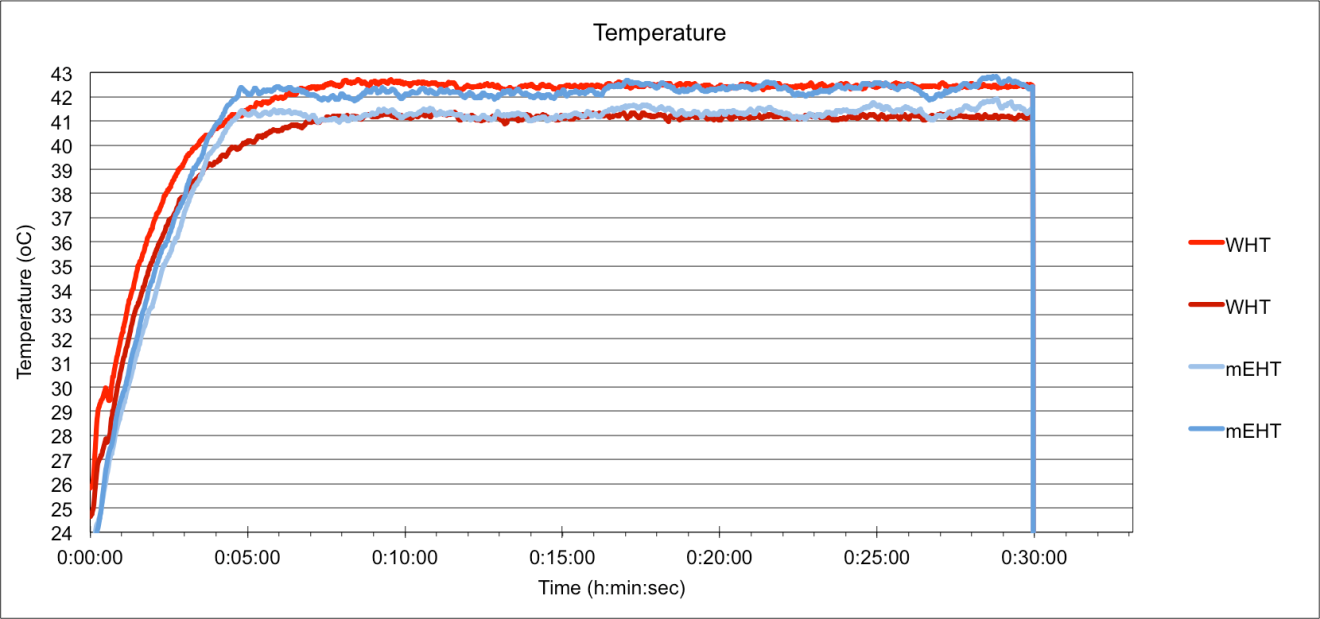


Figure 2. A representative temperature measurement graph during mEHT and (WHT) treatment
